# Scalp surface estimation and head registration using sparse sampling and 3D statistical models

**DOI:** 10.1016/j.compbiomed.2024.108689

**Published:** 2024-06-06

**Authors:** Oded Schlesinger, Raj Kundu, Dmitry Isaev, Jessica Y. Choi, Stefan M. Goetz, Dennis A. Turner, Guillermo Sapiro, Angel V. Peterchev, J. Matias Di Martino

**Affiliations:** a Department of Electrical and Computer Engineering, Duke University, Durham, 27708, NC, USA; b Department of Psychiatry & Behavioral Sciences, Duke University, Durham, 27710, NC, USA; c Department of Neurosurgery, Duke University, Durham, 27710, NC, USA; d Department of Biomedical Engineering, Duke University, Durham, 27708, NC, USA; e Universidad Católica del Uruguay, Montevideo, 11600, Uruguay; f Boston University School of Medicine, Boston, 02118, MA, USA

**Keywords:** Shape estimation, Shape registration, Joint optimization, 3D morphable model, Sparse sampling, Neuronavigation, TMS, EEG

## Abstract

Registering the head and estimating the scalp surface are important for various biomedical procedures, including those using neuronavigation to localize brain stimulation or recording. However, neuronavigation systems rely on manually-identified fiducial head targets and often require a patient-specific MRI for accurate registration, limiting adoption. We propose a practical technique capable of inferring the scalp shape and use it to accurately register the subject’s head. Our method does not require anatomical landmark annotation or an individual MRI scan, yet achieves accurate registration of the subject’s head and estimation of its surface. The scalp shape is estimated from surface samples easily acquired using existing pointer tools, and registration exploits statistical head model priors. Our method allows for the acquisition of non-trivial shapes from a limited number of data points while leveraging their object class priors, surpassing the accuracy of common reconstruction and registration methods using the same tools. The proposed approach is evaluated in a virtual study with head MRI data from 1152 subjects, achieving an average reconstruction root-mean-square error of 2.95 mm, which outperforms a common neuronavigation technique by 2.70 mm. We also characterize the error under different conditions and provide guidelines for efficient sampling. Furthermore, we demonstrate and validate the proposed method on data from 50 subjects collected with conventional neuronavigation tools and setup, obtaining an average root-mean-square error of 2.89 mm; adding landmark-based registration improves this error to 2.63 mm. The simulation and experimental results support the proposed method’s effectiveness with or without landmark annotation, highlighting its broad applicability.

## 1. Introduction

The acquisition and registration of an individual’s scalp surface is important in various research and clinical applications, including brain recording and stimulation. Typically, these steps are supported by a neuronavigation system, which provides the operator with real-time position and orientation information. For example, neuronavigation serves for placing and registering electroencephalography (EEG) electrodes [1], localizing epileptogenic loci and tumors [2,3], and accurately and reproducibly targeting specific brain regions with transcranial magnetic stimulation (TMS) [4]. TMS targets various brain regions including the dorsolateral prefrontal cortex [5,6], the visual cortex [7], the auditory cortex [8], and the cerebellum [9,10]. Neuronavigation with individual head registration can improve the precision and efficacy of interventions [11-15], and accurate estimation of the scalp surface is essential for applications with robotic TMS coil positioning [16].

Factors limiting the accuracy and adoption of such personalized procedures include challenges associated with precisely annotating head keypoints [17]. Moreover, the accurate estimation of the scalp surface required in certain applications, such as robotic coil positioning, necessitates the acquisition of head scans that can be expensive and time-consuming [18].

**Fig. 1.**
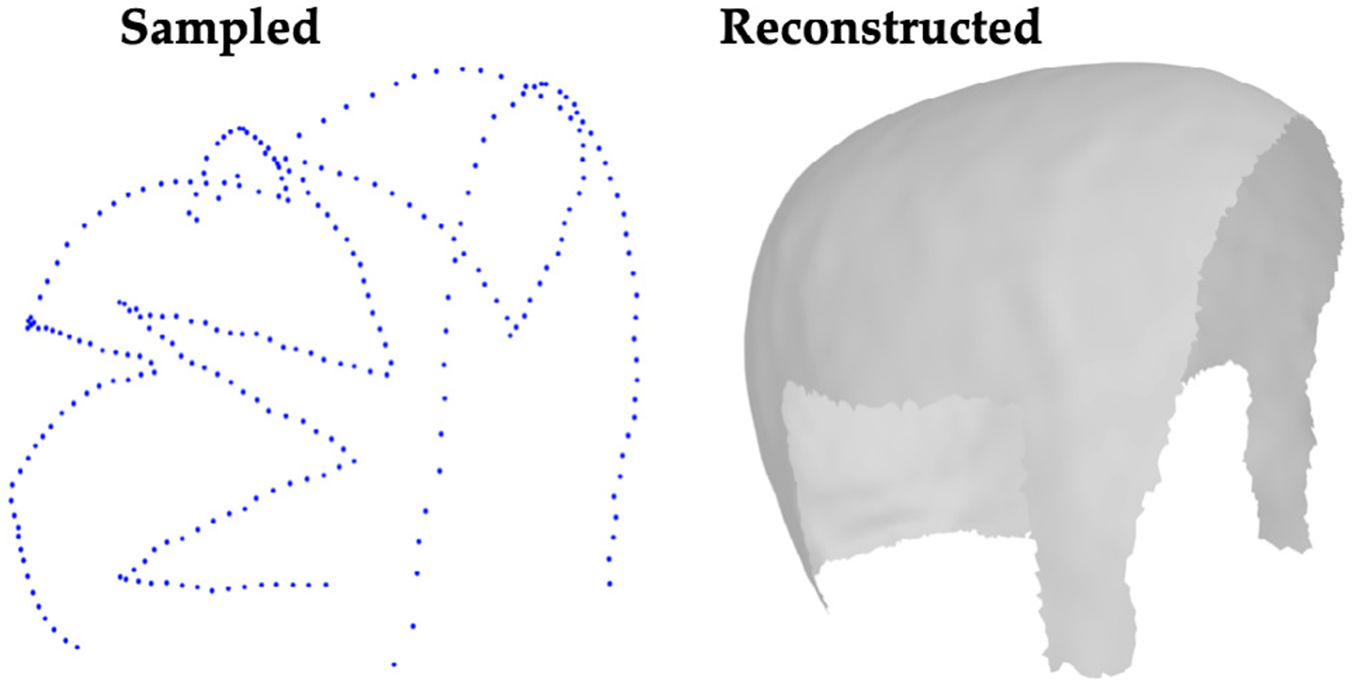
The proposed method leverages a statistical shape model as a prior and a set of sparse surface samples in various input configurations, allowing accurate reconstruction and registration of the scalp surface.

This work takes a step towards overcoming some of these limitations by leveraging recent advances in the field of human 3D head statistical modeling, optimization, and head MRI scan datasets to develop an optimization framework that enables accurate and efficient reconstruction and registration of the scalp surface from sparse samples, as illustrated in Fig. 1. The proposed method is practical and scalable using common tools, e.g., by continuously and sparsely sampling the head surface using existing neuronavigation systems. To infer the shape of regions with no sampling information, we leverage statistical model regularity grounded on information from the sampled regions.

The contributions of this work include: (i) A joint optimization approach for the reconstruction and registration of shapes using sparse and partial data, leveraging statistical models as a prior to estimate unobserved data; (ii) Performing scalable head shape reconstruction and registration, feasible with available neuronavigation tools, while outperforming a commonly used strategy embedded in high-end commercial devices [19]; (iii) Conducting extensive virtual experiments with the proposed method on 1152 publicly available MRIs, using different strategies with various sampling lengths and trajectories; (iv) Demonstrating the robustness and efficacy of our method on 50 subjects whose data was collected in real-life lab conditions with tools commonly used in TMS procedures; and (v) Studying the impact of different sampling factors to inform the optimization of sampling trajectories.

Section 2 of this paper discusses related work and provides additional details to contextualize the present study, Section 3 outlines the main ideas and implementations, Section 4 provides experimental evaluation, Section 5 covers primary findings, and Section 6 summarizes the main contributions as well as the conclusions.

## 2. Related work

Previous work has focused on three related topics: (a) statistical head models, (b) partial shape reconstruction, and (c) head registration and structure estimation.

### Statistical head models.

We focused on the class of statistical shape models referred to as 3D Morphable Models (3DMM). The first 3DMM was introduced in the late nineties by Blanz and Vetter [20]. They proposed a statistical model capturing the variations of 3D facial shape and texture using 200 subjects’ scans. It enabled 3D face reconstruction from 2D images using the controlled manipulation of different components. Several strategies to improve 3DMM followed the ideas introduced by Blanz and Vetter; for example, Booth et al. [21] used data from over 9500 fine-detailed faces to construct the Large Scale Facial Model (LSFM). For several years, work on 3DMM focused on facial reconstruction. Dai et al. [22] addressed this issue and introduced the Liverpool–York Head Model (LYHM), a pioneering 3DMM that includes the cranium and is built on data from 1212 subjects wearing tight-fitting latex caps. Work on partial models was proposed (for the face, ears, head, and so forth), and in 2019 a fundamental work from Ploumpis et al. [23,24] constructed the Universal Head Model (UHM), a detailed 3DMM that combines LSFM and LYHM with other 3DMMs, capturing a fine-detailed complete human head structure from subjects across ages, genders/sexes, and races/ethnicities. Schlesinger et al. [25] assessed the global accuracy of the UHM (built by combining part-based models) validating the model on real 3D complete MRI scans.

### Partial shape reconstruction.

The reconstruction of shapes from partial data is required in many contexts, including various biomedical applications. Amiranashvili et al. [26] reconstructed shapes from various sparse measurements of volumetric medical images based on neural implicit shape representations. Bolkart et al. [27] captured and inferred 3D head shapes in dense correspondence from calibrated multi-view images. Chen et al. [28] proposed a method for 3D cardiac shape reconstruction from sparse and incomplete 2D contour data using a deep-learning architecture. Keller et al. [29] inferred full-body skeletons using regression from real body surface data in an arbitrary pose using a 3D body shape model. Tóthová et al. [30] introduced a probabilistic deep-learning-based approach for organ surface mesh reconstruction from sparse MRI image data. Bernard et al. [31] proposed a method for surface reconstruction from sparse points while modeling the shape distribution using point distribution models and interpreting the available sampled points as ones drawn from a Gaussian Mixture Model, thus trying to maximize their posterior likelihood. Ma et al. [32] trained a neural network to capture the on-surface prior from a dataset during training and leverage it as a differentiable function to learn signed distance functions for unseen sparse point clouds.

### Head registration and structure estimation.

Numerous software packages are commonly used for head registration and scalp surface estimation in transcranial magnetic stimulation (TMS), electroencephalography (EEG), magnetoencephalography (MEG), and other biomedical procedures. A critical step in this procedure is the registration of the subject’s head to an existing head model based on a personalized MRI scan or template. To this end, the operator annotates key fiducial head landmarks such as the nasion and preauricular points [4]. Some systems, including those for robotic TMS coil positioning, require the acquisition of the scalp surface as well [16,33,34]. For accuracy, these existing systems require the use of individual MRI scans and the sampling of the head surface [35]. Common software packages include: MNE-Python [36], SPM [37], NUTMEG [38]. Other commercial solutions, e.g., Brainsight TMS [19] and ANT Neuro visor2 [33], allow the use of landmark annotation to both register and estimate the head structure based on a head template such as CBM152 [39]. The subject’s head anatomy is incorporated in the deformed template for either of these options and, hence, performance does not significantly differ [40]. There has also been an extensive use of anatomical head structure estimation using available measurements. Torres et al. [41] proposed a deep-learning landmark detection method focused on 3D infant head surface shapes. Xiao et al. [42] introduced a method that relies on head-surface sampling to estimate the location of certain neurocranial landmarks. Schlesinger et al. [25] inferred the spatial coordinates of the same landmarks across the neurocranium from observable facial features using deep-learning models. Nguyen et al. [43] predicted the human skull by extracting and computing multiple descriptors of head surface data and providing them to deep-learning models. Wu et al. [44] learned the spatial distribution of the upper part of cranial bones and presented a deep-learning method to infer a complete cranial shape using a partial or damaged shape.

While Nguyen et al. [43] rely on extracting a set of human-crafted features and descriptors and registering all heads into one common coordinate system, our method leverages arbitrary surface samples. In contrast to previous work, we perform automatic co-modeling and registration on a per-subject basis. While Wu et al. [44] leverage most of the head shape to reconstruct a missing part of limited size (up to 35% by volume) and low resolution, our method uses very sparse samples to reconstruct the complete shape at high resolution. Further, most works require the acquisition of 3D volumetric images of the reconstructed shape or use neural networks, which normally require large amounts of human data for training. In comparison, our work does not rely on training or manual annotation, and it can infer and register the head shape using a set of very sparse and unordered spatial samples. We also demonstrate results across the largest sample of subjects.

## 3. Methods

This section details the methodological framework for head shape registration and reconstruction, including the data structures utilized for efficient sampling, the datasets used for training and validation, the joint optimization algorithm for alignment and modeling, the head surface sampling procedure, and experimental validation. Fig. 4 outlines the key stages involved in the proposed methodology.

### 3.1. Data structures and notation

We represent a 3D mesh as a collection of vertices, edges, and triangular faces. Scalar quantities are denoted as lower case variables (x), vectors are represented as bold lower case variables (x), and matrices with capital letters (X). Let us denote the 3D mesh vertices associated with a subject head as $M_{v}=[x_{1}^{T}\;\ldots \;x_{n}^{T}]=[[x_{1},y_{1},z_{1}],\;\ldots \;[x_{n},y_{n},z_{n}]]$, $x_{i}\in R^{3}$; this n×3 matrix is a discrete representation of the subject’s 3D (head) shape; n denotes the spatial resolution of the point cloud formed using the mesh vertices. For each vertex $x_{i}$, we define the associated normal as $p_{i}=[x_{i},y_{i},z_{i}]$, $p_{i}\in R^{3}$. Sets of vertices define the mesh triangular faces, which are being denoted as an m×3 matrix $M_{f}=[f_{1}^{T}\;\ldots \;f_{m}^{T}]=[[f_{1}^{1},f_{1}^{2},f_{1}^{3}],\;\ldots \;[f_{m}^{1},f_{m}^{2},f_{m}^{3}]]$, $f_{i}\in {\left(1\ldots n\right)}^{3}$, while edges are defined as an l×2 matrix and consist of the faces sides $M_{e}=[e_{1}^{T}\;\ldots \;e_{l}^{T}]=[[e_{1}^{1},e_{1}^{2}],\;\ldots \;[e_{l}^{1},e_{l}^{2}]]$, $e_{i}\in {\left(1\ldots n\right)}^{2}$.

#### 3.1.1. 3D morphable models

3D morphable models (3DMM) are statistical shape (and texture) models capable of representing the manifold of an object’s class. In order to compose a well-grounded and meaningful 3DMM, many 3D object scans and samples have to be acquired and aligned. This class of particular models is linear, data are represented by a mean shape of the sampled data, $S_{mean}$, and corresponding principal component vector. These are obtained by applying principal component analysis (PCA) on the aligned training samples, allowing the analysis of their variation by finding the major underlying trends in different scans, each comprising numerous points defining the shape. PCA effectively addresses this data complexity by identifying the directions of greatest variation in the acquired data. Retaining only the most significant variations allows for the rich and comprehensive representation of variations and instances using a significantly reduced set of values. This procedure yields an eigen-decomposition with K components, a set of eigenvalues $e=[e_{1}\;\ldots \;e_{k}]$, $e_{i}\in R$, and eigenvectors $V=[v_{1}\;\ldots \;v_{k}]$, $v_{i}\in R^{n}$. These components are ordered from most significant to least significant in terms of their capability to reflect the object’s class variety. The eigenvectors share a dense correspondence with the mean shape, i.e., faces and edges are stretched between the same vertex indices for different samples, and only vertices change their positions across such samples. This enables the representation of instances $S_{w}$ within the class by multiplying eigenvalues and eigenvectors with customized weights and adding them to the mean shape:

(1) $$w=[w_{1}\;\ldots \;w_{k}],w_{i}\in R,$$

(2) $$S_{w}=S_{mean}+\sum_{i=1}^{k}w_{i}\cdot e_{i}\cdot v_{i}.$$

#### 3.1.2. Graphs

A graph is a collection of nodes and edges stretching between them. Let us denote graph nodes as $G_{n}=[n_{1}\;\ldots \;n_{n}]$, n∈N, and graph edges as $G_{e}$, similar to $M_{e}$. This definition can be extended to weighted graphs, in which edges have numerical values associated with them, $G_{w}=[w_{1}\;\ldots \;w_{L}]$, $w_{i}\in {\left(1\;\ldots \;N\right)}^{2}$. Graphs can be considered undirected, where edges link two nodes symmetrically, or directed otherwise.

A graph walk is defined by the sequence of nodes visited while traversing through the graph.

### 3.2. Datasets and preprocessing

We utilize head surface samples from both existing collections of structural MRI scans and experimental data acquired for this study with a neuronavigation pointer tool.

#### 3.2.1. Mri data

Evaluations are conducted using two publicly available datasets, IXI [45] and ADNI1 Complete 1Yr 1.5T collection (ADNI) [46]. These MRI datasets include 577 and 639 T1-weighted and T2-weighted paired MRI images of subject heads, respectively (see subject statistics in Table 1). While all IXI dataset subjects were considered healthy, approximately 50% of ADNI1 dataset subjects were diagnosed with mild cognitive impairment, 25% were diagnosed with early Alzheimer’s disease, and the remaining 25% were considered elderly controls.

In order to obtain the subject’s ground-truth scalp shape, we first preprocess the head MRI images by segmenting the cranium outer shape and meshing it with the open-source software package SimNIBS 3.2.5, with the headreco head segmentation algorithm [47]. Sixteen and 48 subjects are excluded from the IXI and ADNI datasets, respectively, due to technical issues and unusual image artifacts that impair the integrity of the head shape (examples are shown in the Supplementary Materials, Figure S.1). Consequently, 561 IXI and 591 ADNI subjects are included in the study, adding up to 1152 subjects in total. We aimed to be inclusive of various image imperfections (as shown in supplementary Figure S.2), providing that these do not demonstrate significant artifacts compromising the head representation. Hence, our method should be able to handle noisy samples and certain irregularities.

#### 3.2.2. Experimental surface sampling

Validation of the proposed approach is done over 50 subjects, across different ages, genders, races, and ethnicities, whose data was collected in clinical lab settings (see subject statistics in Table 2). Subjects were recruited from the general population and were not examined for any cognitive or medical conditions. The validation experiments and data collected were performed at Duke University. The study protocol (Pro00109130) was approved by the Duke University Health System Institutional Review Board on May 16, 2023. Written informed consent was obtained from all participants involved in the study.

#### 3.2.3. Preprocessing

In this study, we utilize the UHM as a statistical head shape model. As we focus on scalp shape reconstruction and registration, we first crop the UHM mean shape to include only the scalp surface. In order to crop subjects’ meshes, we align the UHM and the entire subject’s head shape by applying the joint optimization procedure described in the next section. We then utilize the alignment between the two and include all points that are distinctively closer to the UHM scalp than the rest of the UHM shape, as illustrated in Fig. 3. For each subject’s cropped mesh, we construct an associated undirected weighted graph consisting of the mesh vertices as nodes. Graph edge weights, $G_{w}$, are considered to be the Euclidean distances along the mesh edges.

### 3.3. Joint optimization for alignment and modeling

Aligning the subject sampled data and 3DMM scalp point clouds ($S_{sampled}$ and $S_{3dmm}$ respectively) is the most critical component of shape statistical modeling [48,49]. Hence, we propose to solve this important problem via a joint optimization: (i) rigid point clouds alignment (registration), and template deformation (shape modeling). During this optimization procedure, we search for the affine transformation and 3DMM components parameters, which lead to the best alignment between the two surfaces. The first includes (non-uniform) scaling, rotation, and translation parameters, while the latter includes the weights with which we multiply the 3DMM eigenvalues and eigenvectors accordingly, $w_{3dmm}$. A practical algorithmic implementation is possible by leveraging modern deep learning libraries and adapting them to provide powerful and efficient numerical differentiation, as described next.

In recent years, the PyTorch [50] library has become ubiquitous for training neural networks and other deep-learning models. As a result, many frameworks and tools were created, utilizing PyTorch for various uses, including optimization, e.g., BoTorch [51], and 3D data manipulation, e.g., PyTorch3D [52]. Leveraging these state-of-the-art computational tools, we implement our joint registration and modeling optimization framework for reducing the dissimilarity between shapes using PyTorch automatic differentiation mechanisms (i.e., autograd) and PyTorch3D shape manipulation objects and methods (e.g., Transform3d and chamfer_distance). Our optimization framework is end-to-end differentiable and could be applied and optimized for entire ground truth shapes or for collected partial data, which is our main focus.

#### 3.3.1. Joint optimization procedure

We compute the procedure’s initialization point by performing a coarse alignment without using annotated facial landmarks, as often done during registration (see Section 2). We transform the 3DMM mean head shape leveraging subject’s head coarse shape, as captured by sampled data (see Section A.2); let us denote this transformation matrix as $T_{0}$. This transformation will serve as a coarse initialization point for our optimization model described in Algorithm 1.

**Table:** 

**Algorithm 1** Joint optimization procedure
$$\begin{array}{cc} \; & \;\mathrm{Input}:\;S_{mean},S_{sampled},T_{0},\lambda \\ \; & \;\mathrm{Output}:\;S_{reconstructed}\\ \; & \;\;\;\;S_{reconstructed}=S_{mean}\\ \; & \;\;\;\;\mathrm{for}\;i=1,\ldots ,max_{iter}\;\mathrm{do}\\ \; & \;\;\;\;\;\;\;c_{d},c_{n}=Chamfer\;distance(S_{reconstructed},S_{sampled})\\ \; & \;\;\;\;\;\;\;w_{3dmm},T_{i}\leftarrow loss(c_{d},c_{n},w_{3dmm},T_{i-1}-T_{0})\\ \; & \;\;\;\;\;\;\;S_{reconstructed}=S_{mean}+\sum_{k=1}^{num_{comp}}w_{3dmm,k}\cdot e_{k}\cdot v_{k}\\ \; & \;\;\;\;\;\;\;S_{reconstructed}=T_{i}\cdot S_{reconstructed} \end{array}$$

Pseudo code of the algorithm used for aligning and deforming a statistical head shape model to the subject’s samples. loss step is done using an Adam optimizer. Using $c_{n}$ is optional. Early stopping occurs when loss does not decrease over multiple epochs.

The proposed optimization model input consists of the 3DMM mean shape vertices and computed normals (optional), eigenvectors and eigenvalues, the coarse initialization point transformation parameters, as well as the subject’s vertices and their computed normals (optional). In each iteration, we morph and transform the template to match the sampled trajectory points.

#### 3.3.2. Loss function

In order to reduce dissimilarity between shapes, we utilize the Chamfer distance [53]. Since the reconstructed shape is in the form of a 3D mesh, we consider not only vertices distances $c_{d}$, but also their corresponding normals distances $c_{n}$. As Chamfer distance usually quantifies the similarity between shapes of similar spatial resolution and number of data points, it may struggle with quantifying it in distinct asymmetrical cases, e.g., regions where one shape’s spatial resolution is much higher than the other [54], which are common when optimizing using sparse data. To overcome this we consider only distances originating in the sampled points,

(3) $$c_{d}=\frac{1}{|S_{sampled}|}\cdot \sum_{x\in S_{sampled}}\underset{y\in S_{3dmm}}{\text{min}}{\left\|x-y\right\|}^{2}.$$

The same principle applies to normals, we consider the normals similarity associated with the correspondents found during $c_{d}$ computation, $x_{c}=argmin_{y\in S_{3dmm}}{\left\|x-y\right\|}^{2}$. Thus,

(4) $$c_{n}=\frac{1}{|S_{sampled}|}\cdot \sum_{x\in S_{sampled}}1-|\frac{n_{x}\cdot n_{x_{c}}}{\left\|n_{x}\|\cdot \|n_{x_{c}}\right\|}|.$$

An underlying assumption in our approach is that human head samples are drawn from a multivariate Gaussian distribution,

(5) $$S_{sampled}∼𝒩(S_{mean},\Sigma ).$$

Thus, its components are univariate Gaussian distributions. Under the statistical head shape model we utilize, each customized weight which controls the model deformation, $w_{i}$, is independent of other weights and corresponds to a specific trend of variation, as established during the UHM construction [23,24] and added to the model’s mean shape to determine the magnitude of that variation in the reconstructed shape. Hence, the customized weights are distributed from a Gaussian distribution with zero mean, μ=0,

(6) $$w_{i}∼𝒩(0,\sigma^{2}).$$

Shape variations are defined by deviations from the UHM mean shape. These could be quantified using a distance which includes customized weight values, that determine the magnitudes of those variations in the reconstructed shape. To this end, in order to prevent unlikely deviations and template over-fitting, we regularize the Frobenius norm of the 3DMM components’ weights, $w_{3dmm}$.

Let us denote $T_{i}$ as a transformation matrix that is constructed using optimized scaling, rotation, and translation parameters and, as mentioned earlier, $T_{0}$ as a transformation matrix that is constructed using those of the coarse initialization transformation. Regularization is also done over the Frobenius norm of the element-wise differences between transformations, to constrain large deviations from the initialization point. Each of the loss function terms is multiplied by a coefficient to enable control over the different terms. Hence, we aim to minimize

(7) $$reg=\lambda_{3}\cdot {\left\|w_{3dmm}\right\|}_{F}+\lambda_{4}\cdot {\left\|T_{i-1}-T_{0}\right\|}_{F},$$

(8) $$loss=\lambda_{1}\cdot c_{d}+\lambda_{2}\cdot c_{n}+reg.$$

### 3.4. Sparse surface sampling

In this study, two distinct data acquisition modalities are employed to capture surface data used for the reconstruction and registration of head shapes. MRI scans are utilized in Sections 4.1, 4.2, and 4.3. In Section 4.4, a clinical neuronavigation pointer tool is employed to capture subject head surface geometry.

#### 3.4.1. MRI head models

**Fig. 2.**
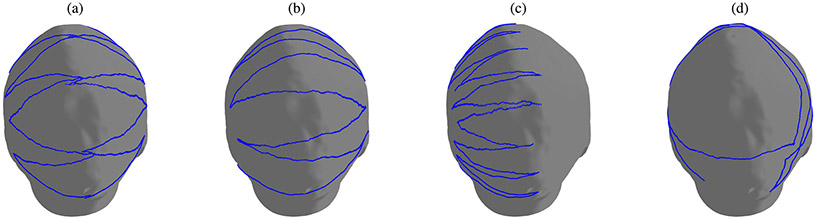
Illustrations of sampling strategies, in blue: (a) side-to-top — continuously moving in a zigzag pattern from the side to the top of the scalp, repeating the movement for each side of the head; (b) side-to-side — continuously sampling the scalp surface moving from side to side in a zigzag pattern, starting from the area in front of the ear, towards the occipital bone on the back of the head; (c) half side-to-top — continuously moving in a zigzag pattern from the side to the top of the scalp, for only one side of the head; and (d) random walk — traversing between vertices chosen randomly.

**Fig. 3.**
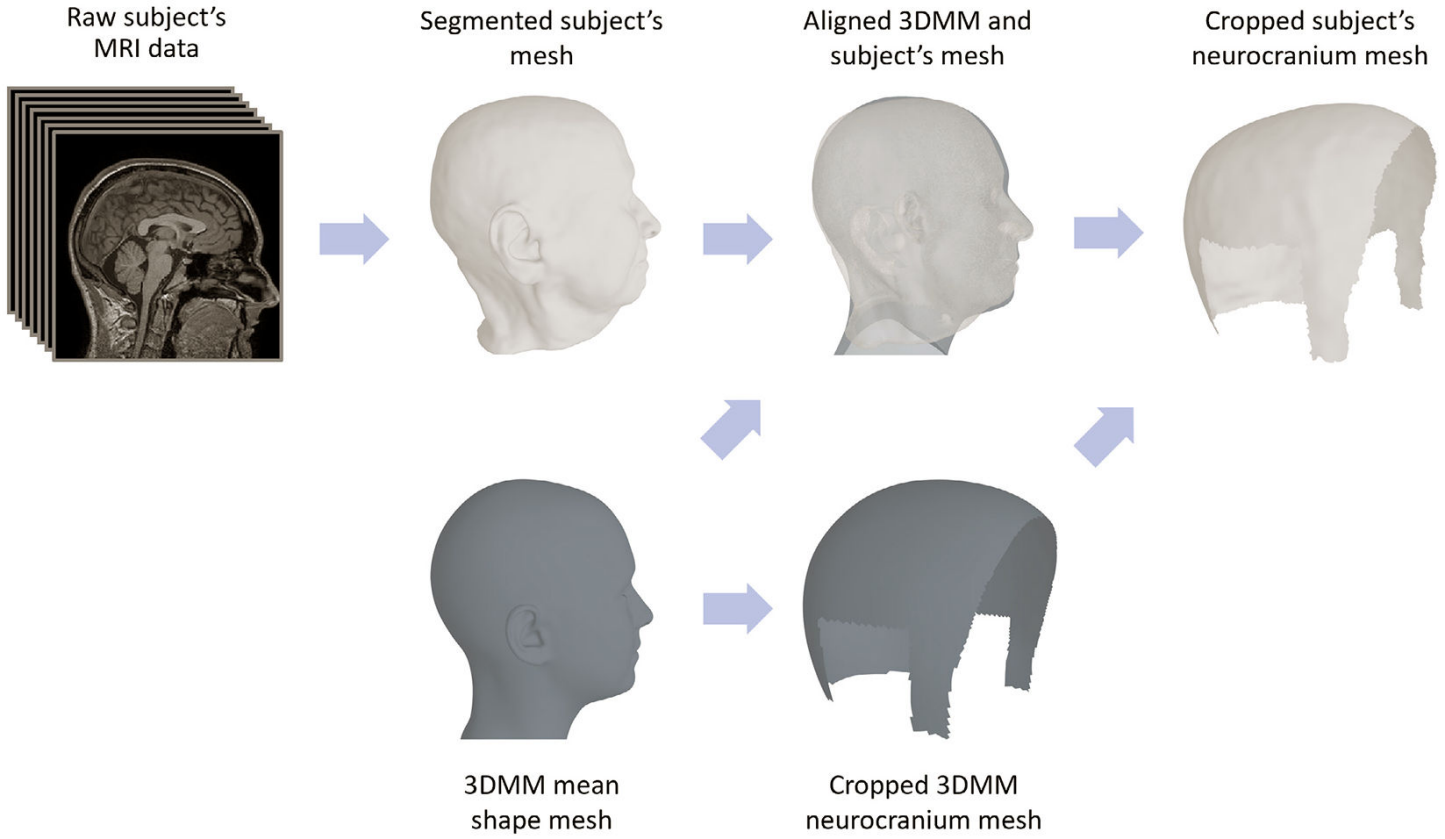
MRI data preprocessing pipeline. Raw subject’s MRI scans are transformed into a 3D mesh, which is segmented into different anatomical regions. Segmented data are inspected for artifacts and external objects that were captured during data acquisition; if present these are manually removed. The 3DMM is aligned and morphed to match the subject’s 3D mesh using the entire face and head shapes. Once alignment is completed, we select the scalp region which is used for contiguous curve sampling.

**Fig. 4.**
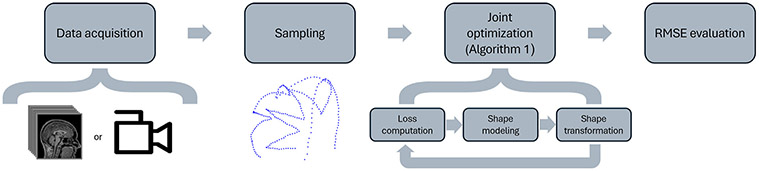
Diagram of the proposed methodology for the reconstruction and registration of head shapes using sparse and partial data. In the first stage, data are captured using either MRI images or stereo camera recordings (). Subsequently, sparse sampling is performed to acquire a subset of data points from the head surface (Section 3.4). Next, Algorithm 1 is employed to align and fit a 3DMM serving as a head template. In the final step, accuracy of the reconstructed head surface is evaluated.

Surface sampling is done by acquiring the spatial coordinates of visited nodes, without using the information of edges connecting those, while walking through the weighted graph associated with the head shape, as described in Section 3.1.2. We present three strategies that allow for well-covering yet efficient sampling of the head: side-to-top, side-to-side, and half side-to-top. In each of them, the probe follows a twisting course over the scalp surface, starting from the area in front of the ear, towards the occipital bone, while traversing the side and top of the head; another strategy includes a random walk over the surface (see Fig. 2). Data acquisition by these strategies is based on the assumption that contiguous sampling is most practical as a consequence of the registration probe movement across the scalp, but this is not necessary, and the sampling can also be non-contiguous. Data points acquired using sampling trajectories encompass on average only about 2% of the sampled shape’s surface. Sampling trajectory normals are estimated, using sampled data only, while making sure they are facing outwards of the head. For each sampled trajectory, we collect its geodesic accumulated length, defined by the sum of edge weights and vertex position displacements.

When sampling using non-random strategies (side-to-top, side-to-side, and half side-to-top), the head is divided into n+1 sections, n denotes the number of steps in the continuous walk (two continuous walks using the side-to-top strategy). Sections are partitioned according to their angle along the curve connecting the frontmost point in the scalp and the backmost point and continuing towards the neck, such that partitions stretch along arcs of equal angles and are ordered along this curve (see illustration in the Supplementary Materials, Figure S.4). An additional internal partition into three sub-sections of each section exists to ensure that sampling curves progress in approximately equal intervals along the arc connecting the frontmost and backmost scalp points. The first step starts in the area in front of the ear; following steps start in the area corresponding to the step number and end in the area corresponding to the step number plus one. These walks are limited to points within their start and end regions.

We conduct experiments using the proposed method over all valid subjects in IXI and ADNI datasets. For each subject, we use 5 unique sampling walks in varying lengths chosen randomly in the range of 1–16 semi-continuous steps; thus, for each sampling strategy we have Numberoftrajectories=5760. Reconstruction and registration are done using the contiguous sampling trajectories, including all vertices they visited. Sampling trajectory lengths are computed by accumulating Euclidean distance between adjacent points in continuous walks. As a benchmark for comparing our approach, we reproduce the registration and reconstruction method using the conventional registration procedure defined by the Brainsight TMS neuronavigation user manual [19], as mentioned in Section 2. This procedure is performed in two different ways; one includes the use of three fiducial head landmarks often utilized during registration (nasion and preauricular points), and four other neurocranial landmarks (topmost, backmost, leftmost, and rightmost scalp points), referred to as extremities; the second, and more common in practice, involves using only the first three landmarks. While the extremities are normally chosen on Brainsight TMS out of a set of approximated points, we choose them precisely by leveraging the alignment described in, thus improving the robustness and reliability of the benchmark shape reconstruction and registration results. Furthermore, choosing this as the benchmark allows us to quantitatively compare our method with its performance over the head surface using the same statistical head model over multiple head MRI scans. For a fair comparison, we use the same head template, UHM [23,24], in both techniques.

#### 3.4.2. Experimental recordings

**Fig. 5.**
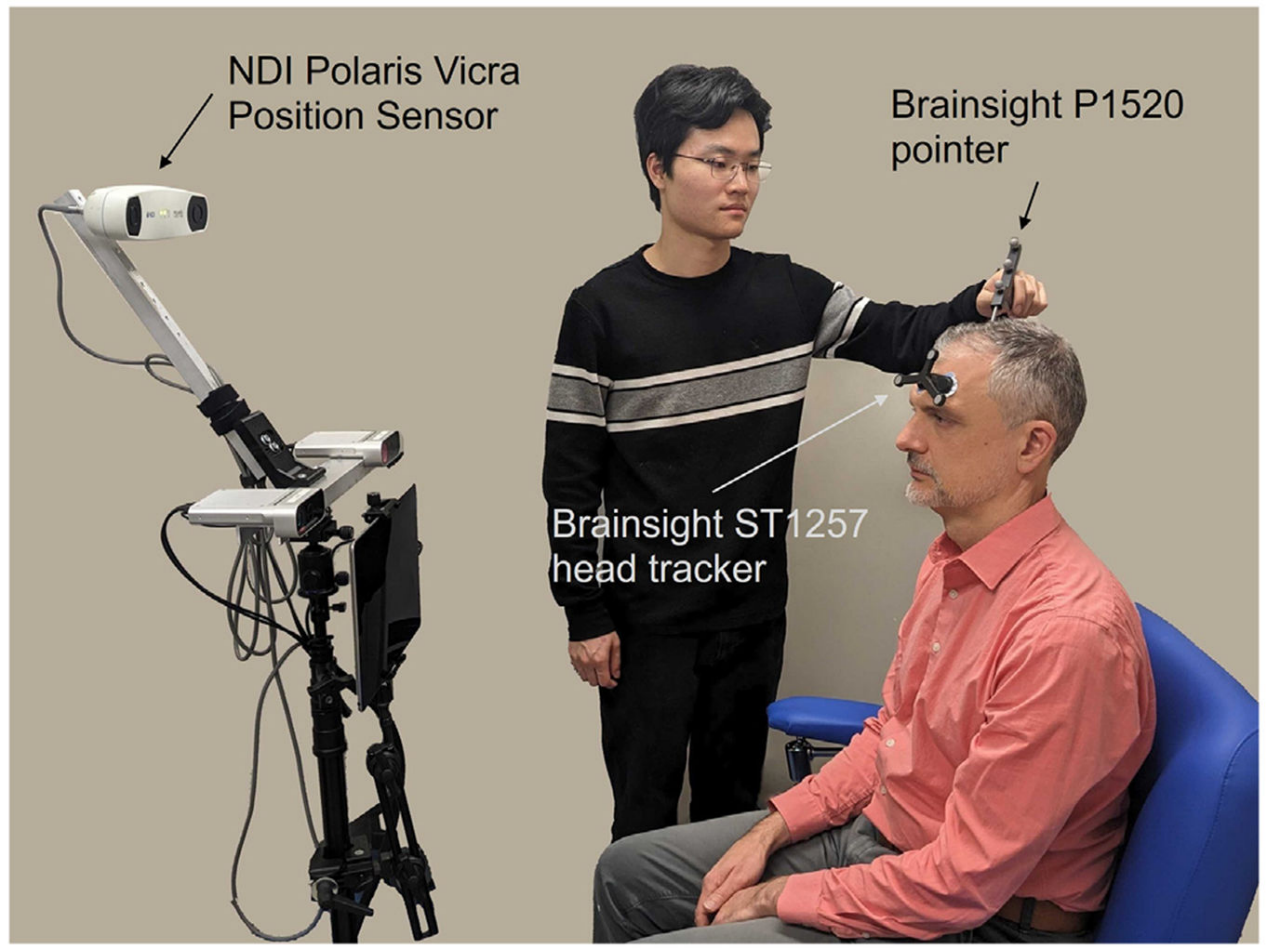
Validation study data collection setup. Illustration of head surface samples being acquired using Brainsight P1520 pointer, with the subject having a Brainsight ST1257 head tracker on. NDI Polaris Vicra Position Sensor (top cameras) is used for capturing and tracking subjects’ head location (by using the tracker position) and samples (by using the pointer’s tip position relative to the head tracker). Both subjects in this photo consented to its use.

Following standard neuronavigation procedures common in TMS sessions, the spatial pose of a pointer (Brainsight P1520, Rogue Research) is captured by an infrared stereotaxy camera (Polaris Vicra Position Sensor, NDI). Subjects wear a tracker (Brainsight ST1257, Rogue Research) attached to their forehead with an adhesive pad, enabling the precise tracking of the position of their head. This configuration allows for surface samples to be acquired by continuously recording the position of the tip of the pointer relative to the head tracker while probing over the scalp surface, as illustrated in Fig. 5. Recording is initiated and terminated under manual control by the operator. Trajectories are manually inspected and any visible artifact is removed; artifacts occurred due to unintentional movement of the head tracker, or the operator unintentionally starting and stopping data collection.

We apply the proposed method to the subject’s samples using partial and sparse data from the scalp surface. As our technique focuses on the subject’s head surface reconstruction and registration without other head scans, we evaluate the method using per-subject cross-validation on the collected data. We split the collected sampling trajectories into twenty segments, using 75% of the sampled trajectories to fit the models and perform registration with the proposed method, and the remaining 25% for validation. This process is repeated 10 times for each subject, with a random selection of validation segments at each iteration, while ensuring bilateral representation and distribution across diverse head regions.

We perform two experiments to reconstruct the head shape of lab subjects. The first mirrors the virtual experiments previously described for the MRI subjects, i.e., without annotation-based registration, as covered in Section 3.4.1. In this experiment, the optimization procedure initialization point is determined following coarse alignment principles similar to the ones mentioned in Section 3.3. In the second experiment, we incorporate conventional fiducial landmarks to guide the registration process (as explained in Section 2). This registration serves as the initialization point for the optimization.

## 4. Results

We run the experiments mentioned in this section with an Adam optimizer [55] and a learning rate of 10^−3^. A machine with NVIDIA GeForce RTX 4090 GPU 24 GB memory conducts the optimizations for $max_{iter}=1000$ iterations with early stopping enabled after reaching convergence. If not stated otherwise, we register and reconstruct subjects’ head shapes with the above-mentioned optimization framework, and $\lambda_{1}\;=\;10$, $\lambda_{2}\;=\;1$, $\lambda_{3}\;=\;1$, $\lambda_{4}\;=\;1$, with spatial distances being measured in centimeters. The impact of different loss term coefficient values is investigated in Section 4.2. Additionally, based on Section 4.2 results, we use the first $num_{comp}=50$ out of the 500 available 3DMM components, capturing 98.2% of the model’s standard deviation as reflected by the sum of these components’ eigenvalues relative to the sum of all eigenvalues.

Under these settings, subject reconstruction and registration takes 38.1 s at most (depending on the number of sampled points). Position displacements are considered to be the Euclidean distances between each of the ground-truth scalp points and their closest neighbor in the reconstructed and registered shape. Reconstruction and registration results are evaluated by measuring position displacement root-mean-square error (RMSE).

### 4.1. Reconstruction and registration

**Fig. 6.**
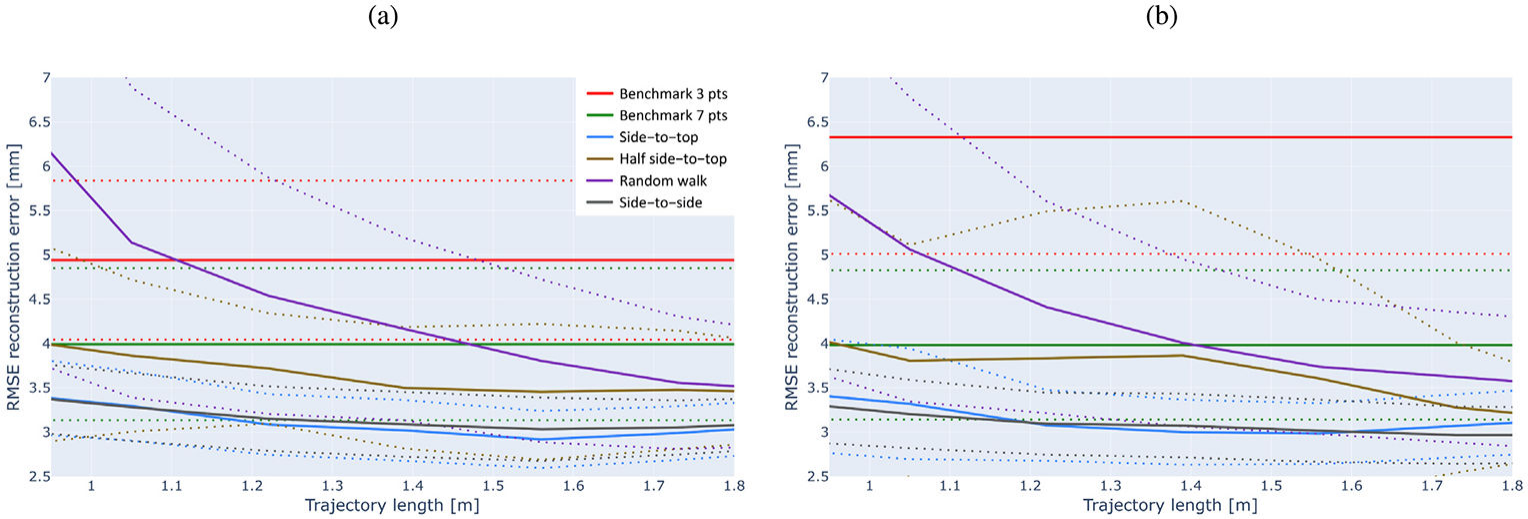
Reconstruction and registration results of the benchmark method (incorporating the use of three or seven landmarks) and the proposed method using different sampling strategies. Plots show results over the convergence region of trajectory length of the proposed method for subjects in the IXI (a) and ADNI (b) datasets. Our method outperforms the benchmark methods without requiring the manual annotation of fiducial landmarks. Results are averaged over continuous intervals. Solid lines represent the mean value and dotted lines the standard deviation.

In this section, we present the results of the experiments detailed in Section 3.4.1. As shown in Fig. 6, the results of our method using all sampling strategies with reasonable trajectory lengths consistently achieve a lower RMSE than the benchmark methods over both tested datasets, regardless of whether the benchmark involves the extremities annotation or not. This also applies to subject scans with certain deformations included in our study, which further confirms the robustness of the proposed method.

#### Impact of sampling trajectories.

As shown in Fig. 6, using side-to-top, side-to-side, and half side-to-top sampling strategies, the errors are significantly lower than the Brainsight TMS results when sampling trajectories are shorter than 1 m over both datasets. In both datasets, when trajectories are about 1 m long, reconstruction results start to converge on average, staying within the same RMSE reconstruction error range. This is the equivalent of a trajectory consisting of approximately 5 side-to-side steps, which can be easily and rapidly performed by the neuronavigation operator.

For the random walk sampling strategy, reported errors outperform Brainsight TMS results involving fiducial landmarks over both datasets when sampling trajectories reach 1.1 m. Further, at trajectory lengths of 1.5 m, our method also outperforms the results of Brainsight TMS leveraging extremities registration as well.

Before reaching convergence, results disperse over a large range of RMSEs, hence their standard deviation (dotted lines in Fig. 6) is much higher than after reaching convergence. Once reaching convergence, both the average of reported RMSEs and their standard deviation are distinctively lower than those of the existing method. That is, our method consistently shows improved results than the common Brainsight TMS method for a variety of sampling paths and strategies. Even when sampling is done in a completely free manner and without any planning, our method still outperforms the benchmark methods when sampled trajectories are on the order of magnitude of 1 m, which can be implemented in practice as we illustrate in experiments reported in Section 4.4.

The results indicate that side-to-top and side-to-side strategies yield lower errors than half side-to-top and random walk strategies across similar trajectory lengths. Side-to-side strategy performance is close to side-to-top strategy, yet it is less practical due to the limitations of the operator’s access to all parts of the subject’s head in one continuous walk.

It can be observed that the longer the sampling trajectory lengths are, the smaller the differences between sampling strategies get. Namely, if one samples long enough, the sampling strategy becomes less relevant. The differences between the various sampling strategies are particularly evident in short sampling lengths, which would be the fastest in practice. These also require the collection of the least amount of data.

### 4.2. Ablation studies

The described joint optimization procedure consists of different parameters, each influencing the outcome. We conduct a comprehensive ablation study to analyze their impact. For simplicity and in order to isolate the effect of the tested component, we conduct ablation study experiments using the side-to-top strategy. This strategy is practical as it allows an operator to sample the subject’s head by covering each side separately while the subject’s head is static and facing the neuronavigation system camera.

#### Number of model components.

The number of template components we use during the optimization is another important factor. A high number of components enables a very rich representation of head instances and various deformations; a low number of components enables faster and more efficient convergence in gradient-descent-based optimizers, such as Adam [55].

#### Loss coefficients.

**Fig. 7.**
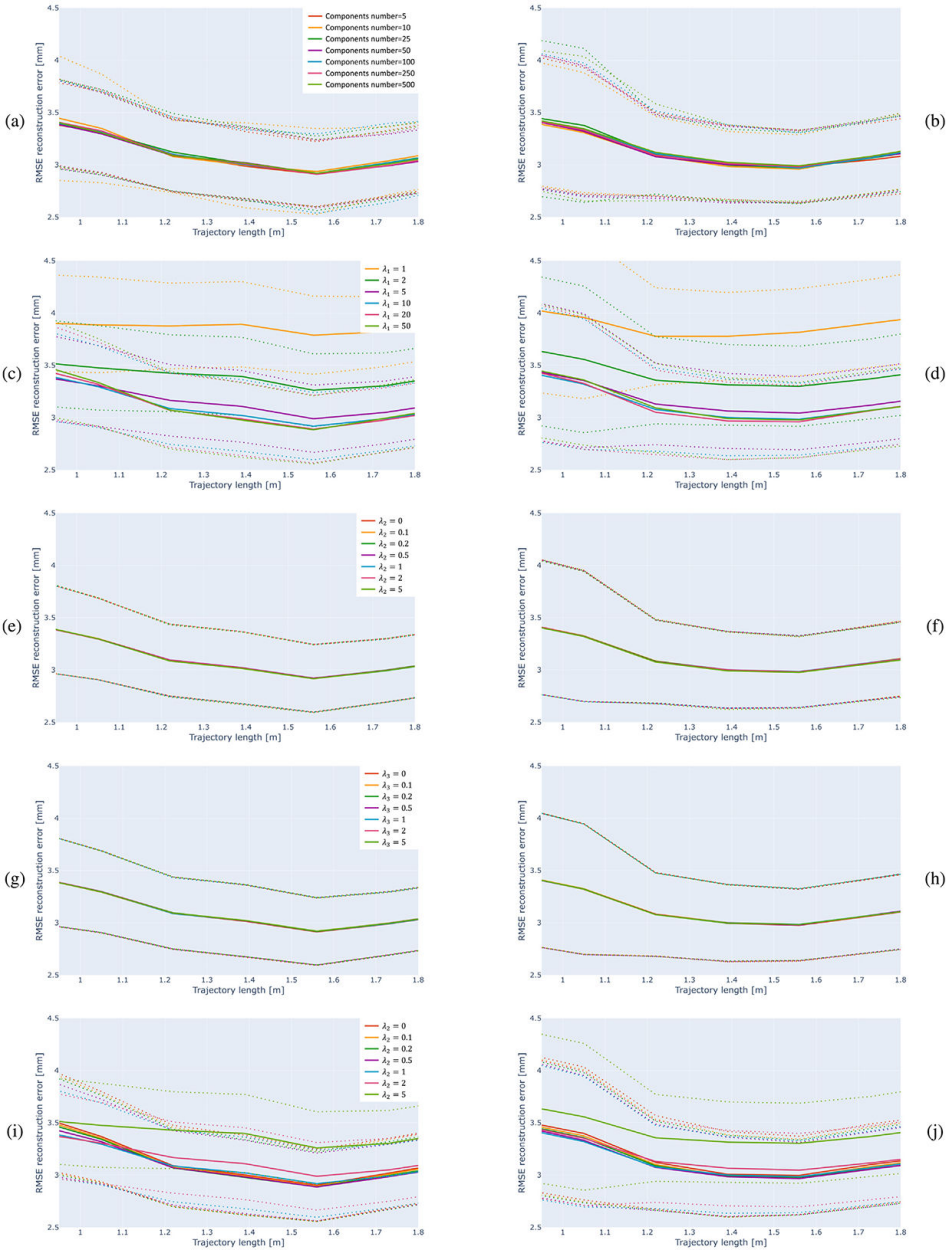
Ablation study results. Results are shown over the convergence region of trajectory length. Left column (a, c, e, g, i) and right column (b, d, f, h, j) correspond to the subjects in the IXI and ADNI datasets, respectively. Top row shows results with varying the number of the model components used while fitting the template to subjects’ head shape. Next four rows show results with varying $\lambda_{1}$ (Chamfer distance coefficient), $\lambda_{2}$ (Chamfer distance normal coefficient), $\lambda_{3}$ (3DMM components’ weights coefficient), and $\lambda_{4}$ (transformation coefficient) values, respectively. Our method consistently outperforms the benchmark methods and shows robustness with respect to the key algorithm parameters.

The loss function, described in Section 3.3, is composed of four different coefficients. Changes in the $\lambda_{1}$ value can affect errors, as it controls the extent to which the optimization tries to minimize the Chamfer distances between point clouds, making the template resemble the ground truth. Lower $\lambda_{1}$ values yield poor results as the resemblance is low, while high values cause the optimization procedure to focus too much on reducing local distances on account of the global shape and other factors. When $\lambda_{1}=0$ (out of scale on Fig. 7), errors are larger than those of the benchmark methods, demonstrating the importance of this factor. Changes in $\lambda_{2}$ have an effect on how much the template surface will be parallel or perpendicular to the ground truth shape along the surface over sampled points based on local information. Parameter $\lambda_{3}$ relates to how rigid the template is during optimization and how flexible it can be in order to morph into other shapes. The range of tested $\lambda_{2}$ and $\lambda_{3}$ values yields similar results. Finally, we penalize excessive transformations, in the form of scaling, rotation, or translation, by multiplying $\lambda_{4}$ by the transformation matrices difference between the initialization point and current ones.

### 4.3. Error estimation

This work’s main focus is scenarios in which the ground-truth head shape, such as from a scan, is not available. In this case, providing an estimated error will allow operators to know when sampling is likely to be sufficiently accurate.

As shown above, estimation outcomes vary over a large range of errors. These depend on many different factors, including sampling trajectory length, sampling strategy, regions visited, and inherent anatomical variations in subject’s head. The results for reconstruction and registration of head shape with sparse surface sampling trajectories in Section 4.1 together with the ablation studies in Section 4.2 indicate that the most impactful factors are the trajectory length and sampling strategy. To gain a deeper understanding of these relationships, we quantitatively evaluate head shape estimation and registration results and their predictability. This analysis involves fitting the estimation error to functions describing different user-controlled parameters, and examining their goodness of fit.

Since the average error plots consist of decreasing graphs with respect to trajectory length, to assess the predictability of this dependence for different sampling strategies we model the RMSE by regression of the trajectory length with an exponential decay function, $f_{r}(x)=a\cdot e^{\left(b\cdot x\right)}+c$, or a polynomial with various number of degrees: $f_{p,m}(x)=a_{0}+a_{1}\cdot x+\cdots +a_{m}\cdot x^{m}$, m = [3,7]. The models are fitted using *Number of trajectories* = 5760 per sampling strategy.

We then use Pearson’s chi-squared statistic [56] for evaluating the goodness of fit, i.e., how well the error model equations fit the data. The chi-squared statistic indicates how predictable the data are by the error model equation, depending on the sampling strategy used. The error predictability may help in planning sampling trajectories given certain limitations, e.g., scalp surface regions that are difficult to sample.

Table 3 shows that the sampling strategies whose results are most predictable according to the length of the sampling trajectory, over both MRI datasets, are side-to-top and side-to-side. They are also the ones which yield the best reconstruction results.

Among the functions examined, a 7th degree polynomial function best describes the empirical results presented in Section 4.1 for all sampling strategies except half side-to-top. An exponential decay function best fits the results for this sampling strategy. Moreover, the exponential decay function shows results very close to the 7th degree polynomial function results for all sampling strategies.

Table 4 provides expected RMSE according to the sampling strategy and length of the sampling trajectory, based on the regression functions that best fit the results according to Table 3. These can help the operator by providing a real-time approximation of the quality of the sampling, allowing for assessment of the adequacy of the currently sampled data. Moreover, by demonstrating consistently lower expected RMSE across different sampling strategies and trajectory lengths, this table provides additional evidence supporting the superiority of the suggested method.

### 4.4. Experimental validation

We validate the ideas and results presented above with the experimental data of 50 subjects recorded using a neuronavigation pointer tool. Based on the investigation in the MRI-based head models and practical considerations, the subjects’ head outer shape is mapped with the side-to-top sampling strategy. Under these practical lab conditions, sampling trajectories of different subjects vary in length and shape. These range from 1.71 m to 5.83 m; in comparison, the average head circumference of an adult human is approximately 0.56 m [57].

**Fig. 8.**
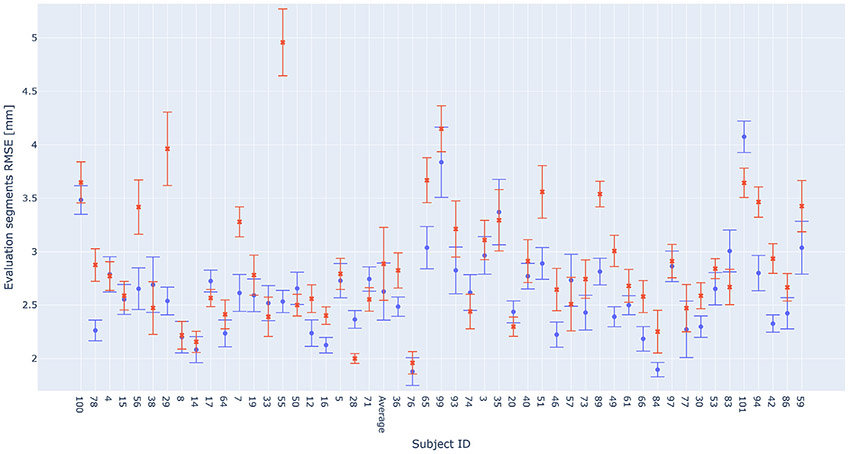
Reconstruction RMSE of data collected from 50 subjects with conventional neuronavigation tools and settings. The RMSE is evaluated over various trajectory validation partitions. The joint optimization procedure initialization point are: (a) the one used by the benchmark and involves using fiducial landmarks, as covered in Section 3 (blue dots), or (b) free conditioned, several centimeters away from the subject’s shape (red X’s). Subjects are ordered according to the length of sampling trajectories used for achieving these results; these range from 1.71 m to 5.83 m. The head surface of several subjects (ID33, ID55, ID56, ID65, ID78, ID89, and ID101) is sampled in a non-contiguous fashion due to their hairstyle which limits the access to the scalp surface over certain regions.

The mean and standard deviation of reported RMSE are presented in Fig. 8, summarizing the findings from both experiments across all cross-validation segments, as described in Section 3.4.2. It can be observed that the results of the two experiments are close to each other in most subjects. As expected, in most cases, the second experiment results, where more data are used, are slightly better, with an average RMSE of 2.63 mm. This confirms that our method enables skipping the registration step which uses more data, requires more time to collect, and advanced knowledge of the head structure, yet reconstructs the head shape with good precision.

Lab subjects’ sampling trajectory may include surface samples very close to each other due to certain repetitions throughout the course of the trajectory. Additionally, their lengths are longer than those of MRI 3D head surfaces with a similar sampling strategy and without using annotation-based registration. Hence, we expect reported errors to be slightly lower from the values computed in Section 4.3. One can notice this is indeed the case, as the average RMSE over lab subjects when not using registration is 2.89 mm, 0.06 mm less than the converged RMSE computed over MRI datasets subjects under similar sampling settings.

## 5. Discussion

To investigate the proposed method for head registration and shape estimation from scalp surface samples, we employed two complementary experiments. First, we conducted various virtual experiments using ground-truth MRI data from 1152 subjects. Second, leveraging the insights gained from the MRI-based simulations, we validated the method’s accuracy through experiments on samples collected from the scalp surface of 50 subjects with conventional neuronavigation tools and settings. In these studies, the proposed method achieves an average reconstruction and registration RMSE of 2.95 mm and 2.89 mm, respectively. Registration using anatomical landmarks annotation further improves the results.

For comparison, the dimensions of a representative TMS motor cortex target are on the order of 15 mm [58]. The cortical target localization error is less than 15 mm and typically 4–8 mm on average [3]. The mean neuronavigation errors are around 5.0–5.7 mm with up to 11.5 mm 95% confidence interval for the complete system including registration, model errors, and tracker movement [59,60]. The mean error between scalp landmarks on subject-specific MRI scans and corresponding individualized head templates employed for neuronavigation is 4.69 mm [61]. Mean TMS coil-to-head coregistration errors fall within the range of 2.2–3.6 mm; these primarily stem from head-to-MRI registration [35].

By analyzing virtual experiment results and factors, we found that the sampling strategy has a significant effect on the scalp reconstruction results, as demonstrated in Section 4.3. The half side-to-top sampling strategy, which deliberately covers only half of the scalp, demonstrates the importance of balanced coverage of the head. Head shape reconstructions using this strategy rely on one half of the desired, asymmetrical shape and tend to align closer to the sampled half. Yet, it still manages to achieve an error significantly lower than the benchmark, and less than 0.60 mm on average above other non-random methods (side-to-top and side-to-side) over the MRI datasets. Thus, it is successfully completing the shape of the other, unsampled half of the head.

The random walk sampling strategy illustrates that basic planning of the sampling route along the head is important but not necessary. One must take into account the fact that due to its randomness, some regions may be sampled more than once in a certain trajectory. This strategy yields higher errors compared to the other non-random methods for similar trajectory lengths over the MRI datasets. However, the random walk also converges to similar performance as the length increases.

The side-to-top and side-to-side sampling strategies achieve results very close to each other over the MRI datasets across different sampling trajectory lengths. Nonetheless, as discussed earlier, the latter is more practical than the former.

The results shown in Sections 4.1 and 4.2 represent the reconstruction of the head shape using sparse surface sampling trajectories on a large number of subjects. Notably, regardless of their length the sampling trajectories comprise a few percent of the total acquired shape, on average only about 2%. To this end, we achieve an accurate reconstruction of the head shape using very sparse sampling.

A potential limitation of our work includes evaluations being done differently over MRI-based data and experimental data collected with neuronavigation tools. The former was evaluated over the entire acquired shape while the latter was evaluated over segments of sampled points. Yet, both help to demonstrate the efficacy of the proposed method in two different scenarios yielding similar errors, even while dealing with noise, operator error, and other factors introduced in real-world settings.

The work presented in this paper is the first to study a new head surface reconstruction and registration technique using sparse surface sampling trajectories and 3D statistical models. In addition, it includes a comprehensive quantitative analysis of the impact different factors have on the method performance.

## 6. Conclusions

This paper proposes a practical method for efficient estimation and registration of the surface of individual human heads, focusing on sampling of the scalp, without the need for individual head scans. The method achieves an average reconstruction and registration RMSE of less than 3 mm in both simulations with MRI scans as well as in experiments using conventional neuronavigation tools.

The proposed method involves joint optimization that leverages recent progress in deep learning algorithmic development, in particular some of its optimization building blocks, to implement an efficient solution for the joint optimization of fine registration and modeling from sparse head samples. An accurate complete head shape can be obtained from minimal sampling by regularizing the problem via rich statistical head models and priors. These software tools are practical and were validated with common neuronavigation hardware and setup in clinical settings.

In contrast to other methods, our method only requires surface samples, which are easy to obtain and do not require the precise annotation of anatomical landmarks. When using the validated side-to-top sampling strategy, for sampling trajectories longer than approximately 52–74 cm, the performance exceeds the benchmark methods. As the trajectory length increases further, the reconstruction errors converge and stay within a small range. Ablation study demonstrates these apply over several different conditions, and the experimental results show our method is robust to perturbations, noise, and other factors in real-world applications. Collectively, the virtual experiments and the lab measurement results support our method’s robustness and consistency.

## Supplementary Material

MMC1

## Figures and Tables

**Table 1:** Subject composition of MRI datasets (as reported for all subjects, including those whose data are unavailable). IXI was collected from healthy subjects, ADNI was collected from memory and cognitively impaired subjects along with normal aging subjects with no signs of cognitive impairment, depression, or dementia. ADNI dataset does not report subjects’ race or ethnicity.

	Sex [# subjects]				Age [years]			Race or ethnicity [%]				
	All	Female	Male	Unreported	Minimum	Mean	Maximum	White	Asian	Black	Chinese	Other or unreported
IXI	577	313	249	15	20	48.7	86.3	76.9	8.2	2.4	2.3	10.2
ADNI	639	267	372	0	55	75.6	89	–	–	–	–	–

**Table 2:** Composition of subjects in lab measurements. Subjects were volunteers from the general population.

Gender [# subjects]			Age [years]			Race [%]					Ethnicity [%]	
All	Female	Male	Minimum	Mean	Maximum	White	Black or African American	Asian	More than one race	American Indian or Alaska Native	Hispanic or Latino	Not Hispanic or Latino
50	27	23	20	28.6	76	38	30	26	4	2	26	74

**Table 3:** Goodness of fit for different models of reconstruction and registration error (RMSE) based on trajectory length and sampling strategy. Lower Pearson’s chi-squared statistic means a better fit, indicating that a model describes the RMSE better. Best and second-best estimates are highlighted.

	Pearson’s chi-squared statistic					
	Exponential decay	Polynomial of degree [#]				
Sampling strategy		Three	Four	Five	Six	Seven
Side-to-top	5.09	14.68	11.94	6.21	5.92	4.99
Side-to-side	5.44	5.39	5.26	5.26	5.33	5.00
Half side-to-top	19.36	27.71	25.80	22.43	19.56	19.66
Random walk	30.43	31.22	30.53	30.06	29.94	29.91

**Table 4:** Sampling trajectories RMSE and 95% confidence interval estimates by sampling strategy and trajectory lengths. Estimates are obtained while considering all MRI subjects. *Number of subjects* = 1152, from different and diverse populations, and multiple sampling trajectories in different lengths for each sampling strategy, *Number of trajectories* = 5760, as described in . 95% confidence intervals are evaluated based on 200 mm long bins centered at each of the mentioned trajectory lengths for the explored sampling strategies; these are computed using the proportional number of instances for the benchmark methods. In accordance with Section 4.1, side-to-top and side-to-side sampling strategies are expected to yield the best results over longer trajectory lengths. Best and second-best estimates are highlighted.

	Lengths [mm]							
Sampling strategy	300	500	700	900	1100	1300	1500	1700
Benchmark with 3 landmarks	5.65 ± 0.23	5.65 ± 0.23	5.65 ± 0.23	5.65 ± 0.23	5.65 ± 0.23	5.65 ± 0.23	5.65 ± 0.23	5.65 ± 0.23
Benchmark with 7 landmarks	3.98 ± 0.17	3.98 ± 0.17	3.98 ± 0.17	3.98 ± 0.17	3.98 ± 0.17	3.98 ± 0.17	3.98 ± 0.17	3.98 ± 0.17
Side-to-top	18.32 ± 2.02	3.80 ± 0.34	3.82 ± 0.11	3.94 ± 0.08	3.10 ± 0.09	2.83 ± 0.05	3.12 ± 0.05	2.96 ± 0.05
Side-to-side	14.80 ± 0.62	6.89 ± 0.29	3.58 ± 0.08	3.33 ± 0.06	3.29 ± 0.05	3.02 ± 0.05	3.05 ± 0.05	2.97 ± 0.06
Half side-to-top	17.26 ± 1.48	5.47 ± 0.45	3.96 ± 0.30	3.76 ± 0.21	3.73 ± 0.13	3.73 ± 0.24	3.73 ± 0.14	3.73 ± 0.26
Random walk	30.74 ± 1.45	14.81 ± 1.05	8.98 ± 0.69	6.08 ± 0.41	4.80 ± 0.23	4.31 ± 0.17	3.84 ± 0.12	3.59 ± 0.12
