# Supplementary material for: Scalp surface estimation and head registration using sparse sampling and 3D statistical models
Source: Comput Biol Med. Author manuscript; Available in PMC 2024 Aug 1. (PMC11265975; doi:10.1016/j.compbiomed.2024.108689)
Supplement: MMC1 [file NIHMS2003543-supplement-MMC1.pdf]

A. Supplementary Materials

A.1. Datasets and preprocessing details

As mentioned in Section 3.1.2, some images are excluded from the MRI datasets due to technical issues and unusual image artifacts that distort significantly the head surface. Examples of such images can be found in Figure S.1. Other images are included although having certain image artifacts, considering these do not disrupt the head anatomy (see examples in Figure S.2).

Some of the segmented head meshes we considered valid during the preprocessing stage include artifacts in the form of excessive parts of the inner skull and planes in the skull cavity. These might later affect alignment with the statistical head model or the cropping of the scalp shape. In some of the valid scans, subject heads are found at a variety of different angles, or include parts of the headrest and chin support that some subjects used during the scan (see Figure S.3). Such spurious objects and artifacts are removed by detecting mesh planar facets and removing vertices found in their 3D minimum bounding box. Subject head surfaces are not smoothed or processed in any way that reduces their fidelity to the original and true shape. In order to fix the angle of the MRI image head, we rotate it such that the spatial coordinate of the tip of the nose is aligned with the head points median value along the x axis (horizontal direction). Most of the subject images we consider valid still include some unusual deformities, mainly in the form of back and head support artifacts, missing parts of the ears, and cropped noses. These do not affect the virtual experiments carried out in this paper.

A.2. Joint optimization details

We compute the initialization point by performing a coarse alignment without using any facial data, as often done during registration (see Section 2). Instead, we leverage the head's coarse shape, as captured by sampled data. First, we transform the 3DMM mean shape to match the general proportions of the samples representing the subject's head, by uniformly scaling it by r_x such that its span across the x axis will match those of the subject's samples

$$r_x = \frac{max(S_{true,x}) - min(S_{true,x})}{f \cdot (max(S_{3dmm,x}) - min(S_{3dmm,x}))}. \tag{S.1}$$

We take into account the subjects' missing ears, usually not being included in the samples representing the head surface, by adjusting the 3DMM span across the x axis, and multiplying it by a factor of $f = 0.9$, based on empirical observations. We also translate the 3DMM mean shape by t so that its point with the highest value over the vertical y axis aligns with the highest point among the subject samples

$$\boldsymbol{t} = S_{sampled}[argmax(S_{true,Y})] - S_{3dmm}[argmax(S_{3dmm,Y})]. \tag{S.2}$$

This is due to the relatively low variation in values at the top part of the scalp.

During the joint optimization procedure, we do not enforce the spatial positions of sampled vertices nor their relative positions, but extrapolate using them.

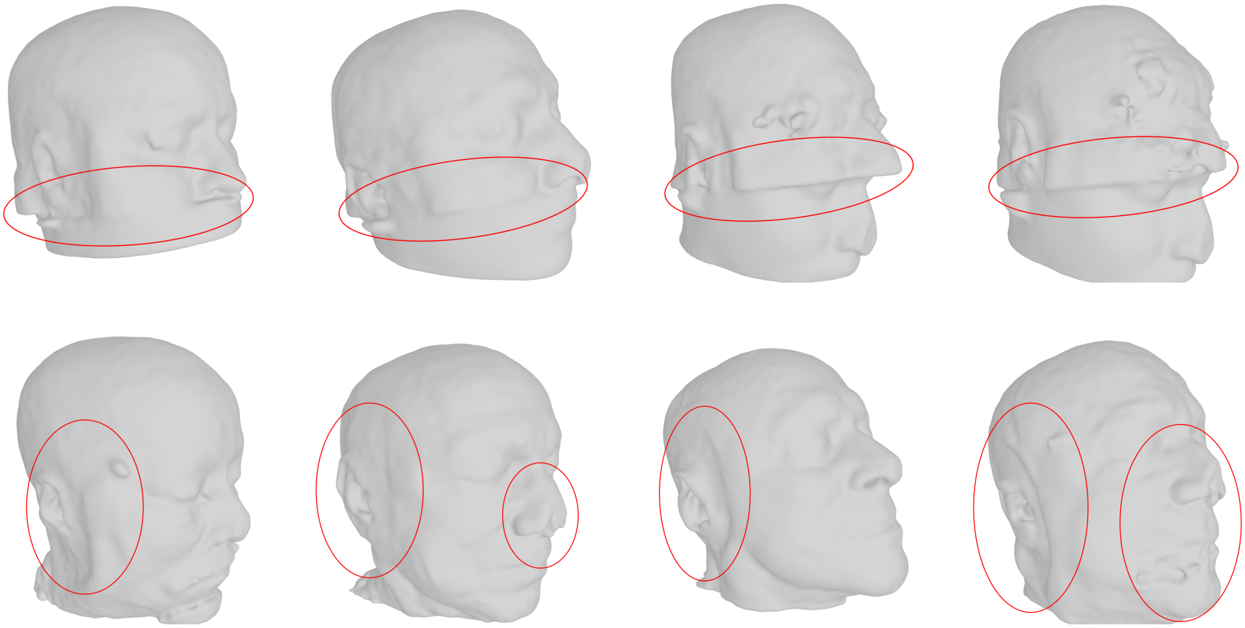

Figure S.1: Examples of subjects excluded due to technical issues and image artifacts that impair the integrity of the head surface (red ellipses). Top row shows IXI dataset examples. Bottom row shows ADNI dataset examples.

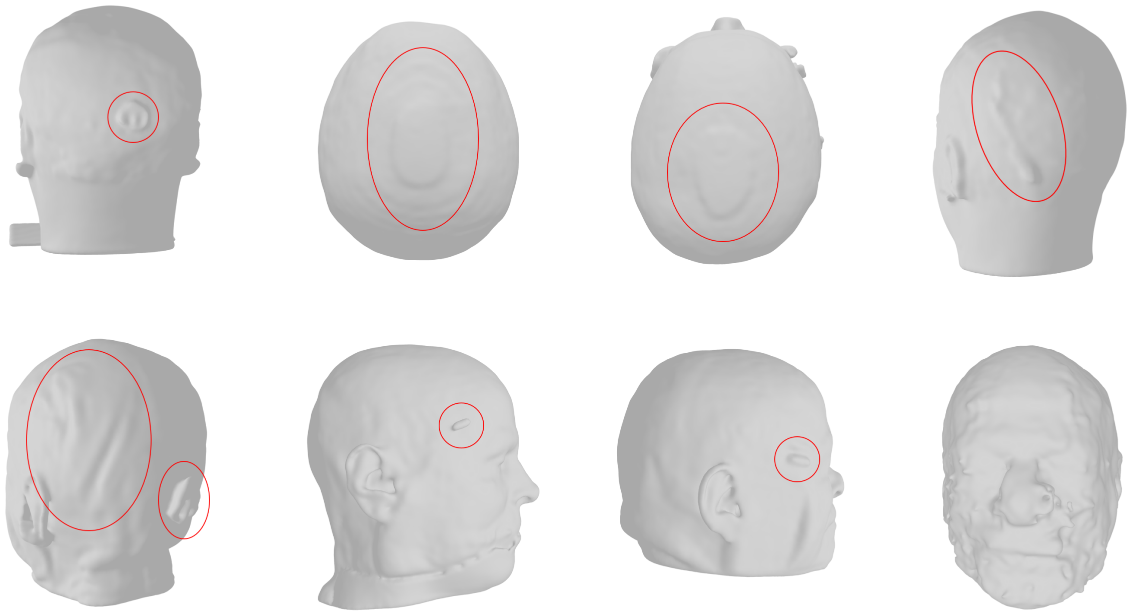

Figure S.2: Examples of subjects included in this study while having slight image artifacts over the head surface (red ellipses). Top row shows IXI dataset examples. Bottom row shows ADNI dataset examples.

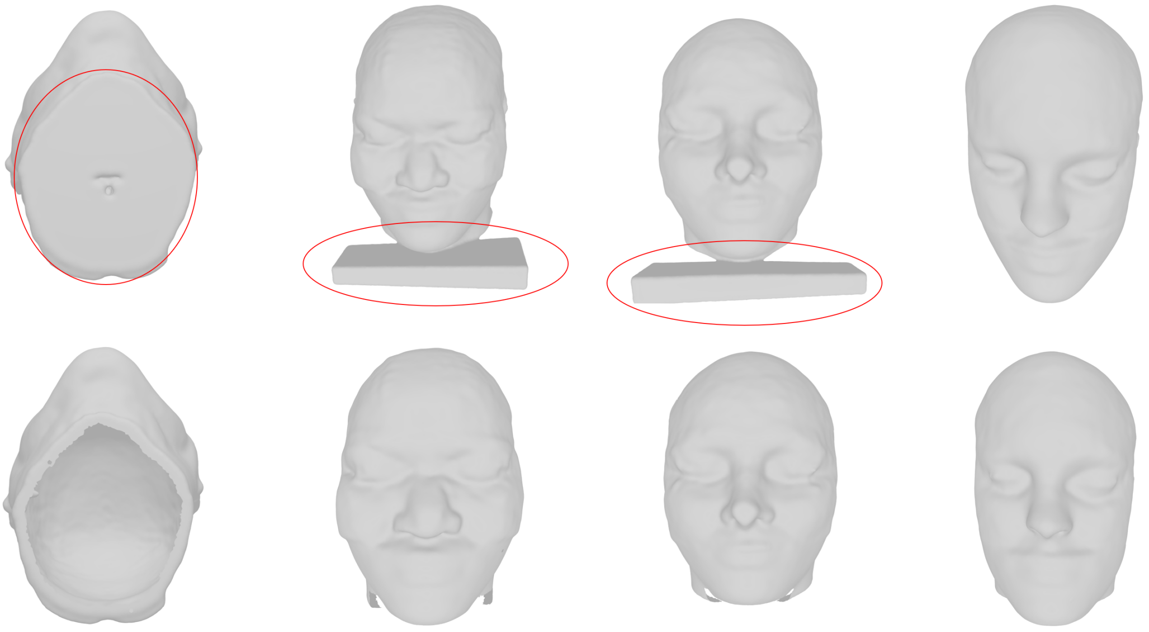

Figure S.3: Examples of the effects of image cleaning we performed. Top row shows images before the data cleaning step. Bottom row shows the corresponding images after they have been inspected and cleaned. Red ellipses in the three leftmost images in the top row identify artifacts or external objects. The rightmost images exemplify the step of image angle correction implemented during cleaning.

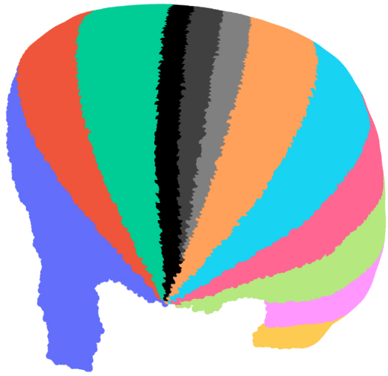

Figure S.4: Illustration of head sections as partitioned when using non-random sampling strategies, with n=9. Grayscale colors demonstrate the internal partition described in Section 3.3.2.
